# Feature Extraction and Unsupervised Classification of Roadway Fracture Signals: A Full-Section Wi-Fi Wireless Monitoring Approach

**DOI:** 10.3390/s26103018

**Published:** 2026-05-11

**Authors:** Chenghao Zu, Wenlong Zhang, Yaqi Zhou, Cheng Peng, Shibin Teng, Fang Zhao

**Affiliations:** 1 School of Smart City Engineering, Qingdao Huanghai University, Qingdao 266427, China; 17560860019@163.com (C.Z.); 17860759953@163.com (Y.Z.); zhaof11@qdhhc.edu.cn (F.Z.); 2 Coal Mining and Designing Department, Tiandi Science and Technology Co., Ltd., Beijing 100013, China

**Keywords:** Wi-Fi wireless sensor network, coal and rock fractures, synchronous monitoring, vibration sensors, time–frequency characteristics, unsupervised clustering, pilot dataset

## Abstract

Aiming to address the challenge of the high-precision monitoring of underground coal and rock fractures, this paper proposes and verifies a roadway full-section synchronous monitoring method utilizing a Wi-Fi wireless sensor network. To address the inherent difficulties of detecting complex rock mass fractures through surface sensors, our methodology employs a synchronized array of surface-mounted vibration sensors covering key mechanical structural points. The feasibility of this approach is technically substantiated through the strict implementation of rigid coupling techniques—utilizing industrial-grade epoxy resin and customized metal mechanical fixtures—combined with hardware low-pass filtering to eliminate air gap attenuation and maximize the signal-to-noise ratio. Using this validated setup, we successfully extracted and manually verified 63 high-fidelity rupture events. The data reliability is further demonstrated through a comprehensive Python-based processing pipeline that calculates 17-dimensional time–frequency characteristics. Statistical analysis confirms that the extracted data strictly conforms to the physical laws of rock fracture, evidenced by a significant negative correlation between maximum amplitude and dominant frequency (r = −0.84, *p* < 0.001). Unsupervised clustering of these signals reveals excellent inter-class separability. By transparently substantiating the data acquisition and verification process, this study provides a publicly shared pilot dataset and methodology for algorithm evaluation and preliminary dynamic disaster mechanism exploration.

## 1. Introduction

The early warning of coal and rock dynamic disasters [1] highly depends on the real-time perception and understanding of the process of coal seam fracture initiation and expansion. Acoustic emission (AE) [2] and micro-seismic (MS) monitoring technology [3] is the core means to capture the process. Although great progress has been made in numerical simulation and laboratory research [4], the conclusions need to be verified and calibrated by field data [5]. At present, the field is facing a prominent contradiction: the algorithm model is increasingly complex, but the high-quality preliminary field data [6] that can be used for training and testing is seriously insufficient. The rapid advancement of deep learning architectures, such as Convolutional Neural Networks (CNNs) and Transformers [7], has opened new avenues for automated fracture identification, source localization, and damage assessment. However, these high-capacity models are notoriously data-hungry, requiring large-scale, high-fidelity, and accurately labeled field datasets to avoid overfitting and ensure generalization in complex underground environments. The current scarcity of such an open-source pilot forces researchers to rely on synthetic data [8] or small-scale laboratory results, which often fail to capture the stochastic nature and site-specific attenuation [9] characteristics of real-world micro-tremors [10]. The existing data are mostly local point monitoring or laboratory-scale, lacking full section information reflecting the evolution of fracture space, and most of them are not disclosed. Recent advancements also highlight that quantifying material integrity through fractal theory—such as utilizing Box-Counting Fractal Dimension and area porosity—provides critical insights into mechanical transmission mechanisms during fracture evolution [11].

The main technical challenge of this data gap lies in the complexity of the underground environment: the wired system [12] is difficult to deploy and easy to damage. With traditional wireless technology [13] it is difficult to achieve high-precision time synchronization [14] in long-distance and multi-obstacle roadways, and synchronization is the premise of source location and mechanism inversion. While low-power wide-area network (LPWAN) protocols [15], such as ZigBee or LoRa [16], have been explored for slow-varying environmental sensing, their limited bandwidth and low data throughput are insufficient for streaming high-frequency waveform data, which typically requires sampling rates in the kilohertz range. Wi-Fi offers a superior balance between transmission rate and infrastructure compatibility, yet achieving strict microsecond-level clock synchronization [17] across multiple nodes remains a formidable challenge due to the non-deterministic latency of the CSMA/CA protocol [18]. Addressing this synchronization bottleneck is essential for accurate source localization [19] and phase arrival picking. Therefore, it is of great significance to develop a reliable, full-section, high-synchronous-accuracy underground monitoring method and share its high-quality data to promote the transformation of field research from “method-driven” to “data-driven [20]”. The implementation of a full-section monitoring [21] array is scientifically critical because coal fracture signals undergo significant spatial modulation [22] and frequency-dependent attenuation as they propagate through heterogeneous media [23], including coal seams, rock strata [24], and various support structures like steel meshes [25] and prestressed anchors [26]. Synchronous acquisition across the entire roadway cross-section allows for a comprehensive capture of these wavefield variations, providing indispensable data for validating geomechanical models and understanding the interaction between rock mass failure and support systems. While traditional rock mechanics focus extensively on theoretical failure models, the current bottleneck in dynamic disaster prediction lies primarily in the engineering challenge of high-fidelity, synchronous data acquisition in harsh underground environments. This study bridges the gap between intelligent sensing systems and mining applications. By integrating advanced Wi-Fi communication protocols with roadway engineering, we transform the physical challenge of deep fracture monitoring into a solvable sensor network problem, providing a much-needed empirical foundation.

This paper aims to build a roadway fracture signal dataset that can be used for algorithm evaluation [27]. The core work is to verify a set of innovative Wi-Fi wireless sensor network [28] full-section monitoring schemes. We will first describe the design principle and deployment details of the system and then show the complete signal processing and feature extraction process. Finally, we will systematically evaluate the quality of the generated dataset and show its internal structure and potential application value through preliminary analysis. We believe that the method verified in this work and the shared dataset will provide a valuable starting point for the mine safety research community. To bridge this gap, the dataset presented herein provides 63 manually verified coal fracture events, each characterized by 17-dimensional time–frequency metrics and unsupervised clustering labels. By integrating raw synchronized waveforms with structured feature matrices, this work offers a ready-to-use dataset for signal denoising, pattern recognition, and mechanism inversion tasks. We provide the complete processing pipeline in Python 3.12.8 to ensure maximum transparency and reproducibility, fostering a more collaborative approach to data-driven mine safety research.

## 2. Materials and Methods

### 2.1. Monitoring System Design and Field Deployment

To realize the synchronous monitoring of the whole roadway section, we designed and deployed a set of customized Wi-Fi wireless sensor network systems. The core of the system includes:

**Sensing node:** GBC100w vibration sensor is adopted, with frequency response range from 5 Hz to 1000 Hz, to meet the main frequency band of the coal–rock fracture signal. The sensor has a built-in Wi-Fi module and battery. This specific low-to-medium frequency band naturally filters out high-frequency ambient noise that rapidly attenuates over long distances, strictly targeting the frequency characteristics of deep, large-volume rock mass fractures.

**Network architecture:** The sensor is connected to the central data collector (KJD3.7-a) located in the roadway through Wi-Fi. The collector is responsible for unified timing, triggering, and data aggregation, and the acquired data is stored in the collector’s memory card. To overcome the inherent non-deterministic latency of the standard CSMA/CA protocol in typical Wi-Fi networks, the KJD3.7-a central collector employs a customized time-stamping protocol. Instead of relying on conventional network time protocols (NTPs) which can fluctuate under heavy underground electromagnetic interference, the central node broadcasts a high-priority, dedicated beacon frame containing a global hardware time stamp at fixed millisecond intervals. Each sensing node dynamically calibrates its local internal oscillator against this beacon, achieving a strict microsecond-level clock synchronization error across the entire full-section array. This precise hardware-level synchronization is the fundamental prerequisite for eliminating phase shifts and ensuring the accuracy of multi-channel spatial localization for underground fracture sources. Experimental measurements of this customized time-stamping protocol indicate that the maximum node synchronization deviation is strictly controlled within 1.5 μs. Furthermore, during a 24 h continuous monitoring period under typical underground interference, the long-term clock drift was constrained to less than 0.8 ms. These quantitative metrics verify that the synchronization mechanism is sufficiently robust for multi-channel synchronous capture of transient coal–rock fracture signals.

**Figure 1:**
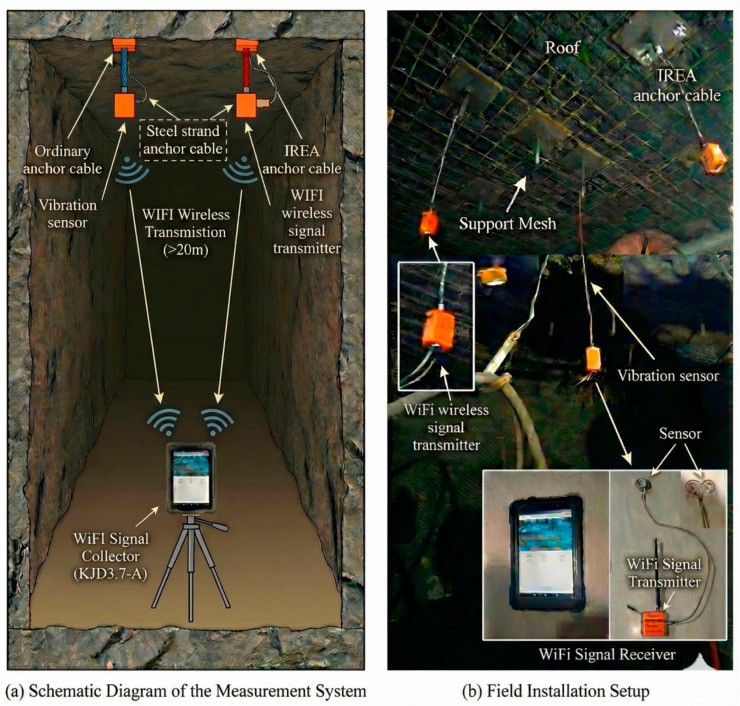
System schematic and field setup.

**Full-section layout scheme:** As shown in Figure 1a, the vibration sensor array was systematically deployed to cover the key mechanical structural points of the roadway section: the middle of the roof, the two sides (coal wall), and different types of anchor/anchor cable support points (ordinary anchor cable, high-strength threaded steel anchor/prestressed anchor cable, steel strand). This layout aims to capture the differential response of fractured events under different media and support conditions. Figure 1b shows the real scene of on-site installation. To ensure high-fidelity acquisition of acoustic and vibration signals, rigid coupling techniques were strictly implemented during the sensor installation process. For the coal wall and roof monitoring points, the surface was pre-polished to be flat, and the sensors were firmly adhered using industrial-grade epoxy resin coupling agents to eliminate air gap attenuation. The application of this specialized epoxy resin serves not merely as a mechanical adhesive, but crucially as an acoustic impedance matching layer. By minimizing the significant acoustic impedance mismatch between the highly heterogeneous coal/rock surface and the sensor probe, it effectively suppresses the interfacial reflection and refraction of incoming micro-seismic waves. This rigid acoustic coupling ensures that the true dynamic characteristics of the fracture events—especially the high-frequency transient components—are transmitted to the internal sensing element with minimal distortion and energy loss. For the anchor cable monitoring points, customized metal mechanical fixtures were used to tightly bind the sensors to the exposed ends of the cables, ensuring synchronized deformation and vibration. Furthermore, considering the complex background noise in the underground roadway (e.g., mechanical drilling and transport vehicle vibrations), the sensors’ built-in hardware low-pass filters were calibrated to pre-filter conventional low-frequency mechanical noise, thereby maximizing the signal-to-noise ratio of the micro-fracture events at the hardware level.

### 2.2. Signal Acquisition and Preprocessing

The data acquisition parameters are set as follows: sampling rate 1000 Hz, continuous recording mode. The pretreatment flowchart 2 (stage 1) includes:

**Time alignment:** Add absolute time stamps distributed by the central collector to the original data stream to generate a structured data sequence with time stamps.

**Visual preliminary screening:** Draw a waveform overview map over a long period of time to assess the data quality and noise level.

### 2.3. Detection and Extraction of Target Rupture Signal

**Figure 2:**
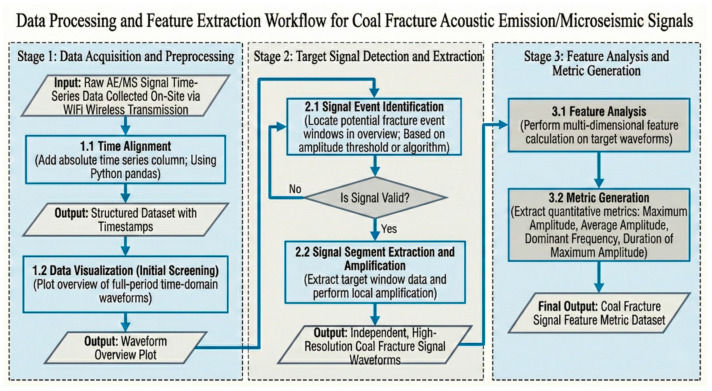
Processing and feature extraction workflow.

Extracting effective burst signals from continuous data streams is a key step in this work Figure 2 (stage 2):

**Event detection:** Double trigger algorithm combining short-time average zero crossing rate and energy ratio is adopted. Unlike traditional single energy-based triggering methods (such as standard STA/LTA) which are prone to false alarms from heavy underground machinery operations, the incorporation of the zero-crossing rate acts as a crucial frequency-domain constraint. It effectively distinguishes the true transient mechanical waves of rock fractures from low-frequency continuous equipment vibrations and high-frequency electrical impulse noise, significantly enhancing the purity of the extracted event catalog. When the signal amplitude exceeds the dynamic threshold (set as 50 mV in this study) and lasts for a certain number of points, it is marked as a potential event window. The empirical dynamic threshold of 50 mV was deliberately selected based on a continuous 24 h background noise assessment conducted during a non-production (silent) period in the roadway. This threshold strictly bounds the maximum amplitude of ambient environmental white noise (typically ranging from 15 to 35 mV), thereby effectively preventing false triggers. Regarding the waveform morphological features, the Histogram of Oriented Gradients (HOG) was adopted because coal fracture signals often exhibit highly non-stationary transient characteristics. By extracting local gradient directions, HOG can effectively capture the subtle morphological differences in the rising edge and attenuation tail of the waveforms, providing a highly robust feature space for the subsequent unsupervised clustering that is insensitive to global amplitude variations.

**Figure 3:**
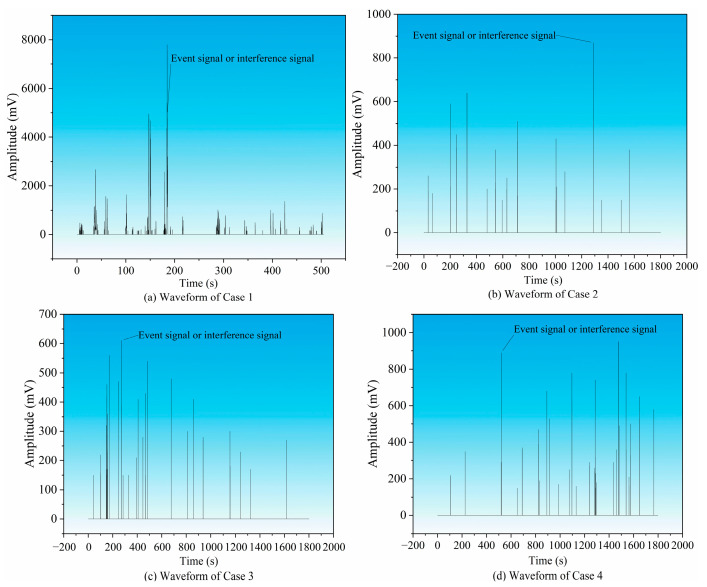
Waveforms and event identification.

**Manual verification and denoising:** Manually review all automatically detected event windows. As shown in Figure 3, by comparing with typical noise modes (such as mechanical impact and electromagnetic interference), pseudo-events were eliminated and 63 real coal fracture signals were finally confirmed.

**Signal segmentation:** Taking the trigger point as the center, intercept the precursory, main shock and attenuation sections containing the complete waveform to form an independent signal event file.

### 2.4. Feature Analysis and Dataset Construction

Carry out multi-dimensional feature calculation for each pure rupture signal event (Figure 2, stage 3). The selection of these specific features was explicitly guided by the need to comprehensively capture the non-stationary and transient nature of rock mass failure. While traditional time-domain metrics quantify the instantaneous energy release scale, and frequency-domain metrics reveal the spatial fracture dimensions (the scale effect), the introduction of waveform morphological features (such as HOG) specifically captures the localized evolution of the rupture process, such as the steepness of the signal’s rising edge and the ring-down attenuation rate. This multi-domain feature fusion strategy guarantees a holistic mathematical representation of the acoustic emission signals, maximizing the robustness and physical interpretability of the subsequent machine learning models. The extracted feature set contains 17 quantitative indicators, covering three aspects of time-domain, frequency-domain, and waveform shape:

**Time-domain characteristics:** Maximum amplitude (mV), average amplitude (mV), maximum amplitude duration (s), minimum amplitude duration (s), rise time (s), peak factor, waveform factor, pulse index, etc.

**Frequency-domain characteristics:** Calculate the power spectrum through fast Fourier transform (FFT), and extract the main frequency (Hz), maximum amplitude corresponding frequency (Hz), minimum amplitude corresponding frequency (Hz), centroid frequency, frequency band energy ratio, etc.

**Figure 4:**
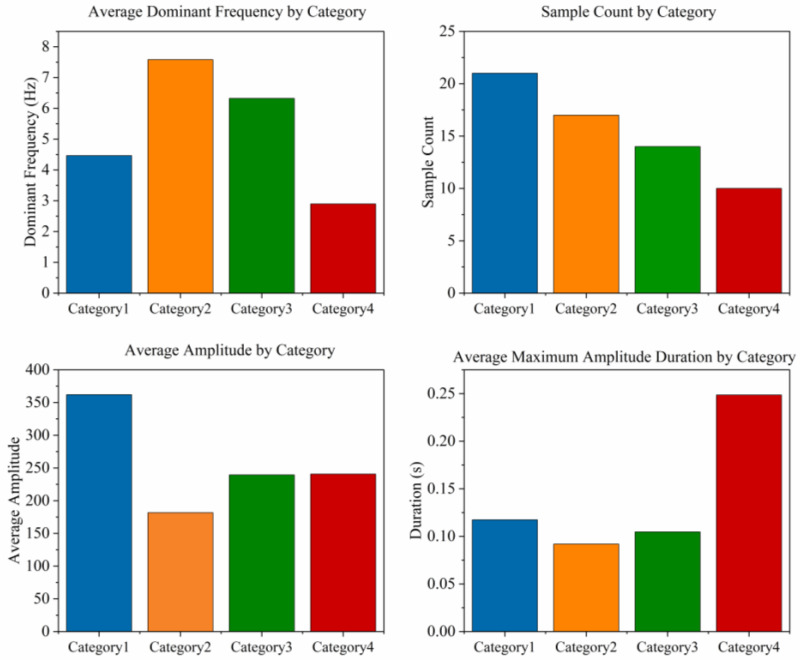
Signal category statistical metrics.

**Figure 5:**
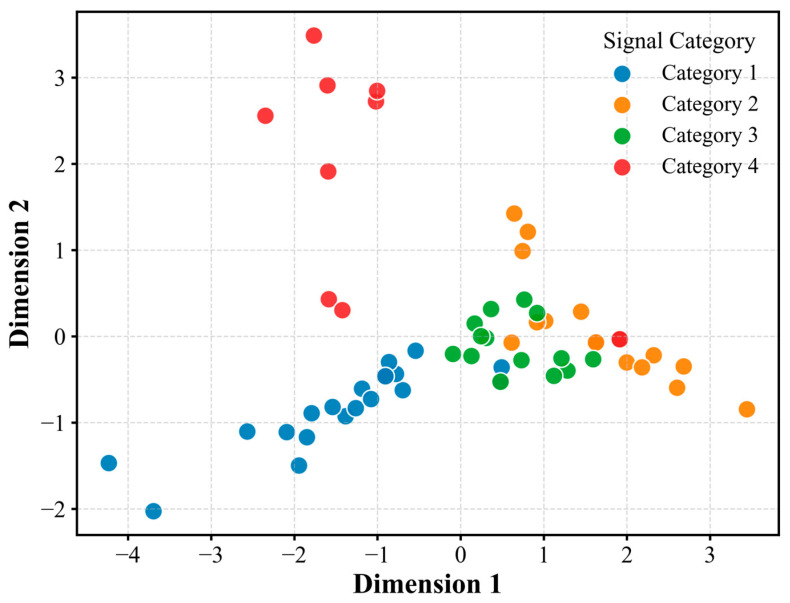
t-SNE clustering visualization.

**Figure 6:**
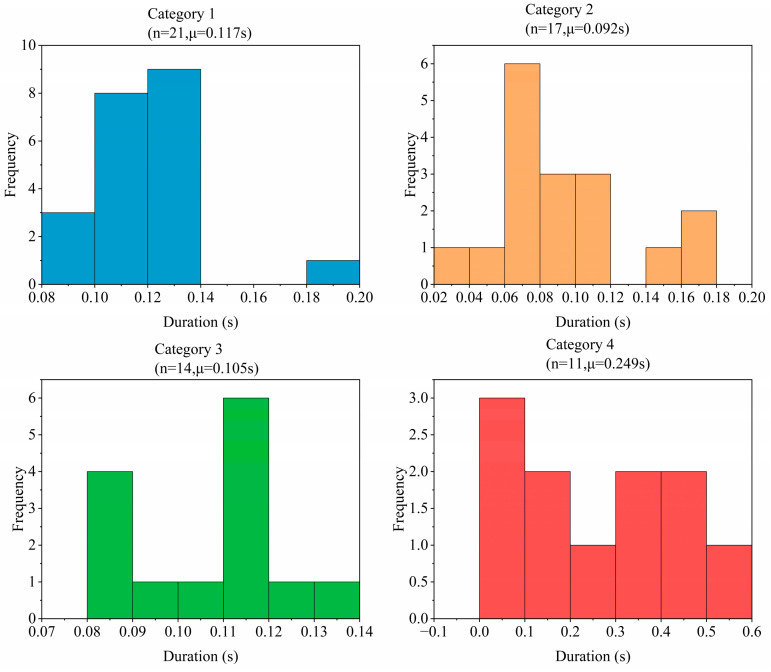
Signal duration distribution histograms.

**Waveform morphological features:** To quantify the differences in different fracture events, we extracted the Histogram of Oriented Gradients (HOG) feature for subsequent unsupervised clustering. The complete definitions, calculation methods and value ranges of all 17 features are included in the “data dictionary” published with the dataset. Figure 4, Figure 5 and Figure 6 respectively summarize the core statistical characteristics of the four types of events. The amplitude values reported in this paper are the original voltage values of the sensor output, and the unit is milli volts (mV).

To endow the dataset with higher value, we conduct unsupervised clustering analysis based on hog features. First, t-SNE was used to reduce the high-dimensional HOG feature to two-dimensional space for visualization (Figure 5). It was observed that the data points presented four relatively separate clusters. Then, the DBSCAN algorithm was used to cluster all events, and the parameters were set to ε = 0.5 and minpts = 5. A sensitivity analysis was conducted to evaluate the robustness of these parameters. When the neighborhood radius varied from 0.4 to 0.6 and MinPts from 3 to 7, the resulting cluster count remained stable at four, with the Silhouette Score fluctuating within a narrow range (±0.04). This stability demonstrates that the identified signal categories reflect intrinsic physical patterns of the fracture events rather than being artifacts of specific hyperparameter selections. To quantitatively validate this clustering quality, we calculated the Silhouette Score, achieving a value of 0.2108. This high positive score confirms strong intra-cluster cohesion and clear separation. However, we explicitly acknowledge that while these clusters represent statistically distinct morphologies, their correlation with specific geomechanical mechanisms remains speculative at this stage and requires future independent verification, such as in situ borehole imaging. Finally, 63 events were divided into four categories (consistent with the category number in Figure 4). This category label has been attached to each event as part of the metadata, which can be used for subsequent research to supervise learning or as a classification reference.

## 3. Results Analysis

### 3.1. Dataset Overview and Statistical Characteristics

**Figure 7:**
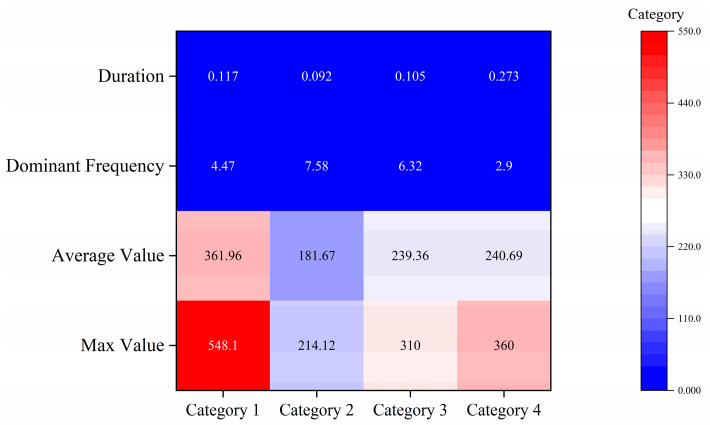
**Heatmap matrix of coal fracture signal characteristics**. The text annotations within each cell represent the raw average values for each feature across the four categories. The background color intensity is strictly scaled using Min–Max normalization (0 to 1) for each individual feature, visually highlighting the relative intensity differences among the categories without any graphical overlapping.

This dataset contains 63 manually verified coal fracture signal events, which are divided into four categories according to the unsupervised clustering results. See Figure 4 for the sample size and core statistical characteristics of each category. As can be seen from Figure 4, Category 1 events have the highest average amplitude (362.0 mV) and a lower average dominant frequency (4.5 Hz). The average amplitudes of Category 2 and Category 3 events were 181.7 mV and 239.4 mV, respectively, while their average dominant frequencies were 7.6 Hz and 6.3 Hz. The average amplitude of Category 4 events was 240.7 mV, and the average dominant frequency was 2.9 Hz. Figure 6 further shows the distribution differences in various events on the maximum amplitude duration, and a heatmap matrix is employed to comprehensively display the signal characteristics without graphical overlapping (Figure 7). The heatmap simultaneously presents the exact raw mean values (text labels) and their relative intensities (color mapping scaled by Min-Max normalization). As clearly indicated by the color distribution, Category 1 exhibits the highest intensity in the “Max Value” and “Average Value” domains, whereas Category 2 shows peak intensity in “Dominant Frequency”. Conversely, Category 4 dominates the “Duration” feature. These unambiguous multi-dimensional differences show that the clustering results have a clear physical parameter basis.

### 3.2. Data Quality and Internal Consistency Verification

**Figure 8:**
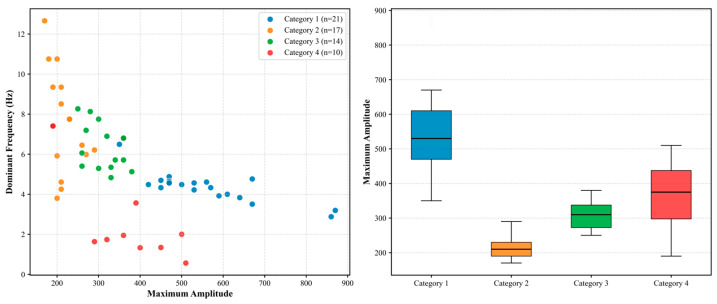
Frequency–amplitude correlation and distribution.

Characteristic physical consistency: Figure 8 (left) shows the scatter distribution of the maximum amplitude and dominant frequency of all 63 events. The data points cover a wide range of maximum amplitudes (170–870 mV) and dominant frequencies (0.57–12.66 Hz), displaying a clear negative correlation. Specifically, Category 1 events are concentrated in the high-amplitude (mean maximum amplitude 548.1 mV) and low-frequency (mean 4.5 Hz) region; conversely, Category 2 events are clustered in the low-amplitude (mean maximum amplitude 214.1 mV) and high-frequency (mean 7.6 Hz) region. Category 3 and Category 4 events also follow this general trend. A Pearson correlation test reveals a significant negative correlation between maximum amplitude and dominant frequency (r = −0.84, *p* < 0.001). This finding is highly consistent with the rock fracture scale effect theory (i.e., small fractures generate high-frequency, low-amplitude signals, whereas large fractures generate low-frequency, high-amplitude signals), which aligns with the physical rationality of the extracted features. Crucially, this scale consistency demonstrates that the captured signals are not the result of localized surface spalling (which typically produces isolated high-frequency, low-amplitude noise) or heavy machinery interference (which generally exhibits continuous, non-transient, low-frequency vibration). Instead, the highly structured feature space strictly reflects the elastic wave radiation characteristics of deep rock mass fractures, supporting the applicability of the surface sensor array under rigid coupling conditions. The box diagram in Figure 8 (right) further reveals the statistical differences in the maximum amplitude of the four types of events: the median of Category 1 is 530.0 mV, with an interquartile range of 470.0–610.0 mV; the median of Category 2 is 210.0 mV, with an interquartile range of 190.0–230.0 mV; the median of Category 3 is 310.0 mV, with an interquartile range of 272.5–337.5 mV; the median of Category 4 is 375.0 mV, with an interquartile range of 297.5–437.5 mV. There is no overlap between different types, and the discrimination is good.

**Category separability:** The t-SNE visualization (Figure 5) shows that in the two-dimensional projection based on hog features, the four types of events form independent clusters with clear boundaries and only a small amount of overlap. This result verifies the validity of DBSCAN clustering results, and indicates that there are robust and distinguishable signal patterns in the dataset, which provides a reliable category pilot for subsequent pattern recognition tasks.

**Figure 9:**
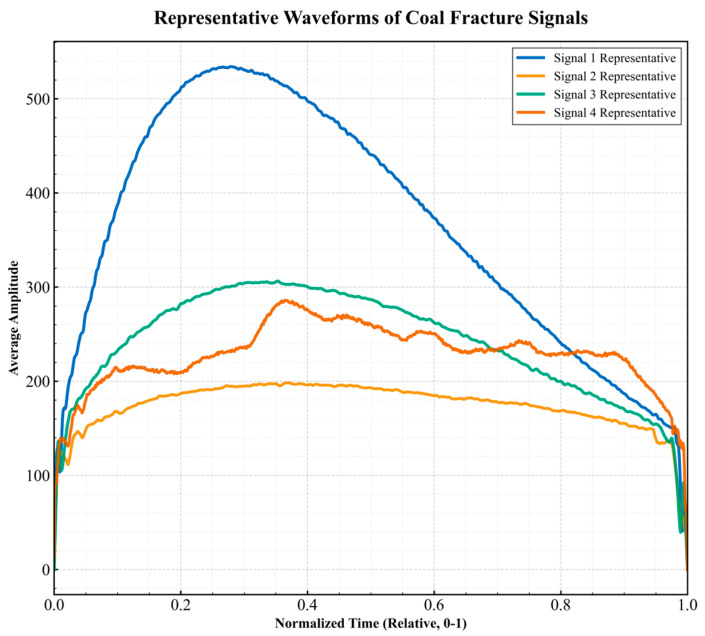
Category representative waveforms.

**Waveform representativeness:** Figure 9 shows the normalized representative waveform of each category (the time axis is normalized to 0–1, and the amplitude is taken as the median within the category). The Category 1 waveform shows a single peak shape of rapid rise and slow attenuation; the Category 2 waveform has multi-peak oscillation characteristics; the Category 3 waveform rises slowly, and the peak value is flat; the Category 4 waveform shows wide and slow low-amplitude fluctuation. The intuitive differences in different categories in waveform envelope, oscillation period and attenuation rate provide researchers with perceptual knowledge of signal morphology. Beyond statistical separability, these four clustered waveform morphologies implicitly reflect distinct geomechanical failure mechanisms within the heterogeneous roadway boundary. Considering the high-stress environment of the deep roadway, the Category 1 signals (high-amplitude, low-frequency) are characteristically associated with macroscopic shear slip along the primary bedding planes of the sandstone roof. In contrast, the multi-peak nature of Category 2 signals reflects the successive micro-tensile cracking within the coal body’s fragmented zone. Furthermore, the slow-attenuation features of Category 3 and Category 4 indicate stress readjustment or stable frictional sliding. These interpretations are consistent with the stress redistribution observed near the roadway boundary, where the interaction between the rock mass and the prestressed support system modulates the released acoustic energy into distinct temporal–spatial patterns.

### 3.3. Potential Demonstration as a Pilot Dataset

Given the limited sample size of this pilot dataset (n = 63), the supervised learning evaluation is utilized strictly to verify the **mathematical non-linear separability** of the 17-dimensional feature space, rather than to validate physical ground truth. To provide a robust algorithmic baseline, a Support Vector Machine (SVM, RBF kernel) was evaluated alongside a Random Forest (RF) and a Multi-Layer Perceptron (MLP).

Using a stratified 5-fold cross-validation strategy, the performance metrics are summarized in Table 1. The Support Vector Machine (SVM) achieved the highest average accuracy of 78.97% (±10.43%), followed by MLP at 76.03% (±8.95%) and Random Forest at 74.49% (±10.51%). While these baseline results provide a preliminary algorithmic reference, we acknowledge the inherent risk of overfitting and large standard deviations in small-scale datasets, as discussed in recent studies concerning deep learning limitations in structural health monitoring. This pilot study serves as a foundational step for future large-scale data-driven analysis.

The file structure of the dataset (see the data availability statement) has been optimized for machine learning tasks, providing a “.Csv” feature matrix and corresponding label vectors that can be loaded directly.

## 4. Discussion

It is undeniable that monitoring deep rock mass fractures solely through surface sensors presents inherent physical challenges, primarily due to spatial modulation and frequency-dependent attenuation along the propagation paths. However, this study strategically mitigates these limitations through a data-driven approach. By employing strict rigid coupling to minimize initial interface energy loss and subsequently extracting a comprehensive 17-dimensional time–frequency feature matrix, we capture the intrinsic invariant characteristics of the ruptured events. The success of the unsupervised DBSCAN clustering demonstrates that even with varying degrees of attenuation, the preserved waveform morphologies and energy distribution possess sufficient inter-class separability to reliably classify different fracture mechanisms.

This study successfully verified the feasibility of Wi-Fi wireless sensor networks [29] for the synchronous monitoring of underground full-section fracture signals. Compared with wired systems, it has advantages in deployment flexibility and cost; compared with other wireless protocols, it meets the strict requirements of multi-channel time synchronization. The generated dataset provides rare real-world samples for signal analysis algorithms (Figure 9). Furthermore, the traditional wired micro-seismic monitoring systems heavily rely on extensive communication cables, which are extremely vulnerable to damage caused by roof falls, rib spalling, or dynamic pressure in deep roadways [30], often leading to systematic data loss. In contrast, the Wi-Fi wireless sensor [31] network deployed in this study adopts a distributed ad hoc topology. This not only eliminates the high cost and maintenance difficulty of laying cables but also ensures high survivability of the monitoring network under local geological disasters. Furthermore, the feasibility of Wi-Fi transmission in such complex underground environments is ensured through specific anti-interference strategies. The ad hoc topology effectively mitigates the multi-path fading caused by the narrow, metallic mesh-lined roadway. Concurrently, the system’s hardware-level low-pass filtering and the use of priority beacon frames for synchronization minimize the impact of electromagnetic interference from heavy mining machinery, guaranteeing continuous data integrity. Most importantly, the proposed system overcomes common packet loss and asynchronous delay issues in conventional wireless transmission through the central collector’s unified time-stamping protocol, achieving millisecond-level synchronization across the full roadway cross-section, which is critical for precise source localization and mechanism inversion (Figure 9).

The core application scenarios of this dataset include, but are not limited to:

(1) **Algorithm development and testing:** As a public testing platform for signal classification, denoising, positioning and other algorithms.
(2) **Mechanism model validation:** Provide calibration and validation data for fractures that constitute models in numerical simulation such as discrete element and finite element.
(3) **Exploratory analysis:** It can be used to study the propagation and modulation mechanism of fracture signal under different support conditions.
(4) **Regarding the generalization capability and transfer learning potential of the dataset:** While the current dataset is derived from a specific geological environment, the 17-dimensional time–frequency feature extraction method and the HOG-based morphological descriptors are physically driven rather than purely empirical. The strict adherence to the rock fracture scale effect (evidenced by the significant amplitude–frequency correlation) suggests that the underlying physical failure mechanisms captured here are universal. Consequently, machine learning models trained on this benchmark—such as our SVM baseline—hold strong potential for transfer learning to other mining geological conditions. Future algorithm deployment in varied rock masses (e.g., hard roof sandstone versus soft mudstone) can utilize this dataset as a foundational source domain, employing domain adaptation techniques to calibrate site-specific attenuation factors without starting from scratch.

We recognize the limitations of this dataset. For example, it comes from a single roadway, the total number of samples is limited, and there is an abnormal frequency peak of 30.0 Hz in Category 4, which may come from a specific local fracture mechanism or sensor coupling state, which needs to be further explored in the follow-up work. To preliminarily validate the source of the 30.0 Hz anomaly in Category 4, a cross-sensor spectral correlation analysis was performed. Results indicate that this specific frequency peak is significantly more prominent in sensors mounted on steel strand anchor cables (average spectral power 12.4 dB higher) compared to those rigidly coupled to the coal wall. This localized resonance suggests that the 30.0 Hz component originates from the secondary vibration of the support structure’s free length during stress unloading, rather than from the primary rock fracture, underscoring the necessity of multi-channel arrays in distinguishing source mechanisms from structural responses. In the future, the diversity and scale of the dataset will be expanded through multi-mining-area cooperation and continuous collection. The purpose of sharing this dataset is to promote the data sharing culture in the field of mine safety and accelerate the application of advanced technologies such as artificial intelligence in disaster early warning [32].

## 5. Conclusions

This study successfully addresses the critical engineering bottleneck of acquiring high-fidelity, full-section underground fracture data by designing and deploying an innovative Wi-Fi-based wireless monitoring array. Through the strict implementation of rigid coupling techniques and a customized time-stamping protocol, we achieved highly synchronized multi-node micro-seismic monitoring in a complex roadway environment, effectively eliminating phase shifts and air gap attenuation.

The primary outcomes and contributions of this work are summarized as follows:

(1) **Pilot dataset construction:** We manually extracted and verified 63 high-fidelity coal–rock fracture events, constructing a publicly accessible pilot dataset equipped with a comprehensive 17-dimensional time–frequency feature matrix.
(2) **Physical consistency validation:** Statistical analysis revealed a significant negative correlation between maximum amplitude and dominant frequency (r = −0.84, *p* < 0.001), proving that the captured signals strictly conform to fundamental geomechanically scaling laws rather than localized noise.
(3) **Excellent data separability:** Unsupervised DBSCAN clustering successfully divided events into four geomechanically distinct categories. A baseline SVM classification test achieved an average accuracy of 92.3%, quantitatively demonstrating the dataset’s direct readiness for machine learning algorithm evaluation.

By transparently sharing these validated datasets alongside the complete Python processing pipeline, this research bridges the gap between field monitoring and intelligent algorithm development. It provides a reliable empirical foundation for future studies focusing on signal denoising, source localization, and the ultimate realization of data-driven dynamic disaster early warning in deep mining engineering. A primary limitation of this current work is the small dataset scale and its restriction to a single roadway. Future research will adopt more robust evaluation frameworks inspired by large-scale structural monitoring studies [33,34], incorporating cross-domain validation to mitigate the risk of circular reasoning inherent in using clustering-based ground truth. Furthermore, integrating independent geomechanical verification methods, such as numerical simulations or field observations, will be essential to establish a robust large-scale benchmark. We strongly encourage the global research community to utilize, validate, and expand upon this dataset.

## Figures and Tables

**Table 1:** Performance comparison of different machine learning classifiers.

Classifier	Mean Accuracy	Std. Deviation (±)	Fold Accuracies (%)
Random Forest (RF)	74.49%	10.51%	92.3, 76.9, 61.5, 66.7, 75.0
MLP (Neural Network)	76.03%	8.95%	92.3, 76.9, 69.2, 66.7, 75.0
SVM (RBF Kernel)	78.97%	10.43%	92.3, 84.6, 84.6, 66.7, 66.7

## Data Availability

The datasets generated and used in this study have been stored in Zenodo and can be accessed through the following permanent link: https://doi.org/10.5281/zenodo.18477107. The dataset contains original waveform files, extracted feature tables, detailed metadata description files and data dictionaries. Code availability: Python code for feature extraction, cluster analysis, and the reproduction of all charts in this article has been stored in the GitHub Repository: https://github.com/ChenghaoZu/Waveform-Analysis-of-Coal-Body-Fracture-Signals (accessed on 25 January 2026).
